# A Comparative Analysis of *Vibrio cholerae* Contamination in Point-of-Drinking and Source Water in a Low-Income Urban Community, Bangladesh

**DOI:** 10.3389/fmicb.2018.00489

**Published:** 2018-03-19

**Authors:** Jannatul Ferdous, Rebeca Sultana, Ridwan B. Rashid, Md. Tasnimuzzaman, Andreas Nordland, Anowara Begum, Peter K. M. Jensen

**Affiliations:** ^1^Department of Microbiology, University of Dhaka, Dhaka, Bangladesh; ^2^Section for Global Health, Department of Public Health, Copenhagen Center for Disaster Research, University of Copenhagen, Copenhagen, Denmark; ^3^International Centre for Diarrhoeal Disease Research, Dhaka, Bangladesh; ^4^Institute of Health Economics, University of Dhaka, Dhaka, Bangladesh

**Keywords:** *Vibrio cholerae*, drinking water, O1/O139, non-O1/non-O139, household, point-of-drinking, source water

## Abstract

Bangladesh is a cholera endemic country with a population at high risk of cholera. Toxigenic and non-toxigenic *Vibrio cholerae* (*V. cholerae*) can cause cholera and cholera-like diarrheal illness and outbreaks. Drinking water is one of the primary routes of cholera transmission in Bangladesh. The aim of this study was to conduct a comparative assessment of the presence of *V. cholerae* between point-of-drinking water and source water, and to investigate the variability of virulence profile using molecular methods of a densely populated low-income settlement of Dhaka, Bangladesh. Water samples were collected and tested for *V. cholerae* from “point-of-drinking” and “source” in 477 study households in routine visits at 6 week intervals over a period of 14 months. We studied the virulence profiles of *V. cholerae* positive water samples using 22 different virulence gene markers present in toxigenic O1/O139 and non-O1/O139 *V. cholerae* using polymerase chain reaction (PCR). A total of 1,463 water samples were collected, with 1,082 samples from point-of-drinking water in 388 households and 381 samples from 66 water sources. *V. cholerae* was detected in 10% of point-of-drinking water samples and in 9% of source water samples. Twenty-three percent of households and 38% of the sources were positive for *V. cholerae* in at least one visit. Samples collected from point-of-drinking and linked sources in a 7 day interval showed significantly higher odds (*P* < 0.05) of *V. cholerae* presence in point-of-drinking compared to source [OR = 17.24 (95% CI = 7.14–42.89)] water. Based on the 7 day interval data, 53% (17/32) of source water samples were negative for *V. cholerae* while linked point-of-drinking water samples were positive. There were significantly higher odds (*p* < 0.05) of the presence of *V. cholerae* O1 [OR = 9.13 (95% CI = 2.85–29.26)] and *V. cholerae* O139 [OR = 4.73 (95% CI = 1.19–18.79)] in source water samples than in point-of-drinking water samples. Contamination of water at the point-of-drinking is less likely to depend on the contamination at the water source. Hygiene education interventions and programs should focus and emphasize on water at the point-of-drinking, including repeated cleaning of drinking vessels, which is of paramount importance in preventing cholera.

## Introduction

Cholera is a life-threatening disease with an estimated 2.9 million cases annually in 69 cholera-endemic countries, including Bangladesh (Ali et al., 2015). A recent review indicated that, in Bangladesh, around 66 million people are at risk for cholera, with an estimated incidence of 1.64 per thousand persons (Ali et al., 2015). In Bangladesh alone, the estimated annual number of cases is 109,000, with a three percent case fatality rate (Ali et al., 2015). Toxigenic and non-toxigenic *V. cholerae* can cause cholera and cholera-like diarrheal illness and outbreaks. *V. cholerae* has more than 200 serogroups based on variations in the “O” antigenic lipopolysaccharide (LPS). Cholera toxin-producing serogroups O1 and O139 have been shown to be the etiological agents of epidemic cholera (Kaper et al., 1995). Non-O1/non-O139 and non-toxigenic *V. cholerae* O1 strains, harboring a range of accessory virulence factors, can cause diarrheal diseases (Morris et al., 1984) and sporadic localized cholera outbreaks (Saha et al., 1996; Faruque et al., 2004; Pang et al., 2007) hence emphasizing the importance of research on both toxigenic and non-toxigenic *V. cholerae*. Accessory factors that can cause diarrheal diseases are repeats-in-toxin (*rtxA*) (Lin et al., 1999; Chow et al., 2001), non-O1 (NAG-ST) and O1 (O1-ST) heat-stable enterotoxins encoded by the *stn* and *sto* genes, respectively (Ogawa et al., 1990; Dalsgaard et al., 1995; Theophilo et al., 2006), hemolysins encoded by the *hlyA* gene (Zhang and Austin, 2005; Karlsson et al., 2013), transcriptional activator (*toxR*) (Waldor and Mekalanos, 1994), hemagglutinin protease encoded by *hap* (Silva et al., 2006; Mohapatra et al., 2009), ADP ribosylating exotoxin (*chxA*) (Awasthi et al., 2013), the type VI secretion system (T6SS) (Unterweger et al., 2012), a novel type III secretion system (T3SS) (Dziejman et al., 2005; Shin et al., 2011), and mannose-sensitive hemagglutinin subunit A encoded by *mshA* (Watnick et al., 1999).

*V. cholerae* can survive in nutrient limited drinking water for long periods of time in a viable but non-culturable state (VBNC) (Colwell, 2009) and can actively exert its infectious capability when in the human intestine (Colwell et al., 1996). This phenomenon poses serious risks to human health due to its non-detectability of VBNC cells by existing culture methods resulting underestimation of colony forming units (CFU) count of viable cells. *V. cholerae* can adapt to and persist in unfavorable environments, such as in conditions of nutrient deprivation and fluctuations in salinity and temperature, and can resist predation by heterotrophic protists and bacteriophages by adopting this unique survival strategy of the VBNC state (Ravel et al., 1995; Colwell et al., 1996; Carroll et al., 2001; González-Escalona et al., 2006; Thomas et al., 2006; Jubair et al., 2012; Mishra et al., 2012). Bacteria remain alive, metabolically active and can express virulence factors in this VBNC state; for example, *V. cholerae* can express *tcp* encoding a toxin co-regulated pilus (Krebs and Taylor, 2011) and the cholera toxin gene (*ctxA*) (Mishra et al., 2012). *V. cholerae* can exert its infectious properties when resuscitation occurs in human and animal digestive tracts (Colwell et al., 1996; Asakura et al., 2007; Senoh et al., 2010). In nutrient limited environments, *V. cholerae* can enter a starvation state in which cells are non-growing but culturable (Colwell et al., 1996; Thomas et al., 2006) and can survive for prolonged period of time (i.e., >700 days) (Jubair et al., 2012). Furthermore, both pathogenic and non-pathogenic *V. cholerae* can attach to abiotic surfaces, i.e., borosilicate glass (Watnick et al., 1999) and can survive in fomites in a VBNC state for more than 7 days (Farhana et al., 2016).

Cholera is endemic in Dhaka city (Patel et al., 2012), and low-income urban communities are particularly vulnerable to cholera and diarrheal diseases due to lack of hygiene and access to clean drinking water (Rafique et al., 2016). Drinking water is considered as one of the primary routes of cholera transmission in Bangladesh (Colwell et al., 2003; Huq et al., 2005; Akanda et al., 2009; Jutla et al., 2011). A recent study in Dhaka city established the association of cholera pathogen and its virulence in drinking water from households with confirmed or suspected cholera case patients (Rafique et al., 2016). There is, however, no known comprehensive evaluation of the burden of *V. cholerae* in source and point-of-drinking water in households in a cholera endemic community. Point of use or household water treatment can be an effective intervention in the prevention of diarrhea (Fewtrell et al., 2005). The World Health Organization has recognized that household water treatment and safe storage can provide rapid and significant health impacts (http://www.who.int/water_sanitation_health/publications/2011/9789241548151_toc.pdf). Therefore, investigating the contamination of drinking water in a population at risk for cholera will be useful to developing specific interventions to protect high risk populations from cholera and cholera-like illnesses. Studies that have investigated water quality at the point of use have focused primarily on water treatment, i.e., filtration, chlorination, flocculation, and solar disinfection of water stored in households (Clasen, 2015; Taylor et al., 2015). Few studies have investigated the microbiological water quality at the point of consumption/drinking (i.e., the quality of water in a drinking vessel immediately before consumption) (Rufener et al., 2010). The aim of this study is to conduct a comparative assessment of the presence of *V. cholerae* between point-of-drinking water and source water and to investigate the variability of virulence profile using molecular methods of a densely populated low-income settlement of Dhaka, Bangladesh.

## Methods and materials

### Study design

The study was conducted in Arichpur, located in Tongi Township of Dhaka, Bangladesh. Arichpur is an urban community with an area of 1.2 km^2^, population density of more than 100,000 residents per km^2^, and approximately 129,000 residents living in 29,000 households (Azman et al., 2015). Residents of this area use water from two types of communal pumps: “WASA (Water Supply and Sewerage Authority) pump” installed by the municipal government and connected to households through underground networks of pipes, and/or “submersible pump” installed by individuals or groups of residents and connected to households through over ground networks of pipes. The area around the pumps is not usually protected with a wall and floor made of concrete. These pumps extract water at a depth of approximately 75–140 m.

### Data collection

A total of 477 households were enrolled in this study. Water samples were collected both at the point-of-drinking and at the source in each study household during routine visits at 6 week intervals from September 2014 to October 2015. Depending on the availability of the caretaker (i.e., the female or male family member who spent the most time in the house), point-of-drinking samples were taken from the drinking vessels (i.e., a mug, glass, bottle, jug, or pitcher) that household members used to drink water. Samples from sources were taken from the communal water source point used by each study household. On average, 20 samples were collected at each weekly visit from point-of-drinking and sources. Caretakers were asked if they treated the water (i.e., boiled, filtered, added alum, etc.) prior to consuming the drinking water. The water samples from sources were taken directly from taps attached to the communal pumps. In the absence of such a tap, samples were collected from taps attached to the nearest closed over-ground reservoir that was connected to the pump. The coordinates of sample collection sites (households and communal sources) were obtained using a global positioning system (GPS). Q-GIS software was used to locate the sites on a Google map.

### Sample collection and enrichment

Each sample contained 100 mL of water that was collected in sterile bottles and transported in a cool box to the Environmental Microbiology Laboratory, University of Dhaka, within 2–4 h of collection. Aliquots of water were added to 10 mL of alkaline peptone water (APW), enrichment medium (1 L distilled H_2_O, 10 gL^−1^ peptone, 10 gL^−1^ sodium chloride; pH 8.5) followed by incubation at 37°C for 18–24 h (Alam et al., 2014).

### Extraction of total DNA and confirmation of *V. cholerae*

After overnight incubation, DNA was extracted from 1 mL of each enriched culture using the method described by De Medici et al. (2003). The presence of *V. cholerae* in water samples was confirmed by detection of the *V. cholerae* species-specific gene *ompW* (Nandi et al., 2000) by PCR. Due to the non-detectability of VBNC cells by existing culture methods, PCR was chosen to reliably detect all forms of *V. cholerae* (both VBNC and culturable).

### PCR reaction mix and primer sequences

*V. cholerae* virulence genes were detected in 143 samples found positive for the *V. cholerae* species-specific gene *ompW* using PCR. A total of 22 *V. cholerae* virulence genes were selected for detection. PCR was performed using an MJ Research PTC-200 Peltier Thermal Cycler (Mexico). The 25-μL reaction mixture contained 2 μL of 10× PCR buffer, 20 mM MgCl_2_, 0.4 μL of 10 mM deoxynucleoside triphosphates (dNTP) mix (Thermo Scientific, USA), 0.1 μL of 5 U Dream Taq DNA Polymerase (Thermo Scientific, USA) per μL, and 1.25 μL of each 25 μM primer (Tag Copenhagen A/S, Denmark). Sequences of the primers and target genes and their amplicon sizes are presented in S1 Table.

Real-time PCR was performed to detect the *V. cholerae ctxA* and *rtxA* genes using an Applied Biosystems StepOne (48-well) Real-Time PCR system. Real-time PCR was used as it provides higher sensitivity and specificity compared to conventional PCR. The fluorogenic probe and primer set (Tag Copenhagen A/S, Denmark) targeting the *ctxA* and *rtxA* genes are described in S2 Table. The formula of reaction mixture and cycling conditions for detection of *ctxA* gene were maintained as per supplier's instruction. The 25-μL reaction mixture containing 12.5 μL 2× TaqMan Universal Master Mix II with UNG (Applied Biosystems USA, with AmpliTaq Gold DNA Polymerase, dNTPs, ROX passive reference, Uracil-N glycosylase), 2.5 μL of each 100 nM of primer, 2.5 μL of 250 nM probe, and 5 μL of template. To detect the *rtxA* gene, a reaction mixture (25 μL) containing 12.5 μL 2× Power SYBR green PCR master mix (with a propriety version of ROX dye), 2.5 μL of each 100 nM sense and antisense primer, 2.5 μL of DEPC-treated H_2_O, and 5 μL of template DNA was used. *V. cholerae* O1 N16961 genomic DNA was used as a positive control, and PCR grade water was used as a no template control for PCR screening.

### Data analysis

The proportions of samples positive for *V. cholerae* in point-of-drinking and source water were calculated. Logistic regression test was employed to examine the association of *V. cholerae* (and virulence genes) between point-of-drinking and sources, treated and non-treated water, drinking vessel type and all the virulence genes. We also examined the association of *V. cholerae* by logistic regression analysis of a set of stratified samples of point-of-drinking water and their linked sources that were collected within 7 days (before/after 7 days) of interval from each other.

### Ethics statement

The Ethical Review Committee (ERC) of icddr,b, Bangladesh reviewed and approved the study protocol. Informed written consent for collecting samples was obtained from caretaker of each household for “point of use” and from pump operator for “source” water.

## Results

A total of 1,463 water samples were collected: 1,082 from the point-of-drinking and 381 from the 66 sources for the 388 enrolled households. Most of the households used mugs (249/388), and/or glasses (195/388), and/or small bottles (75/388) to drink water. Drinking water was treated in 24% (93/388) of the households, and the majority of these households reported boiling (77/93) as the mode of treatment. Twelve households out of 93 reported filtration and three households reported both “boiling and filtration” as the mode of water treatment. Among the 66 water sources for these households, there were three communal “WASA pumps” installed by the government and 63 “submersible pumps” installed by individuals or groups. Of the 66 sources, 31 had direct taps attached to the communal pumps, and 51 had taps attached to the reservoir connected to the pumps.

### *V. cholerae* in “point-of-drinking” and “source” water

**Figure 1:**
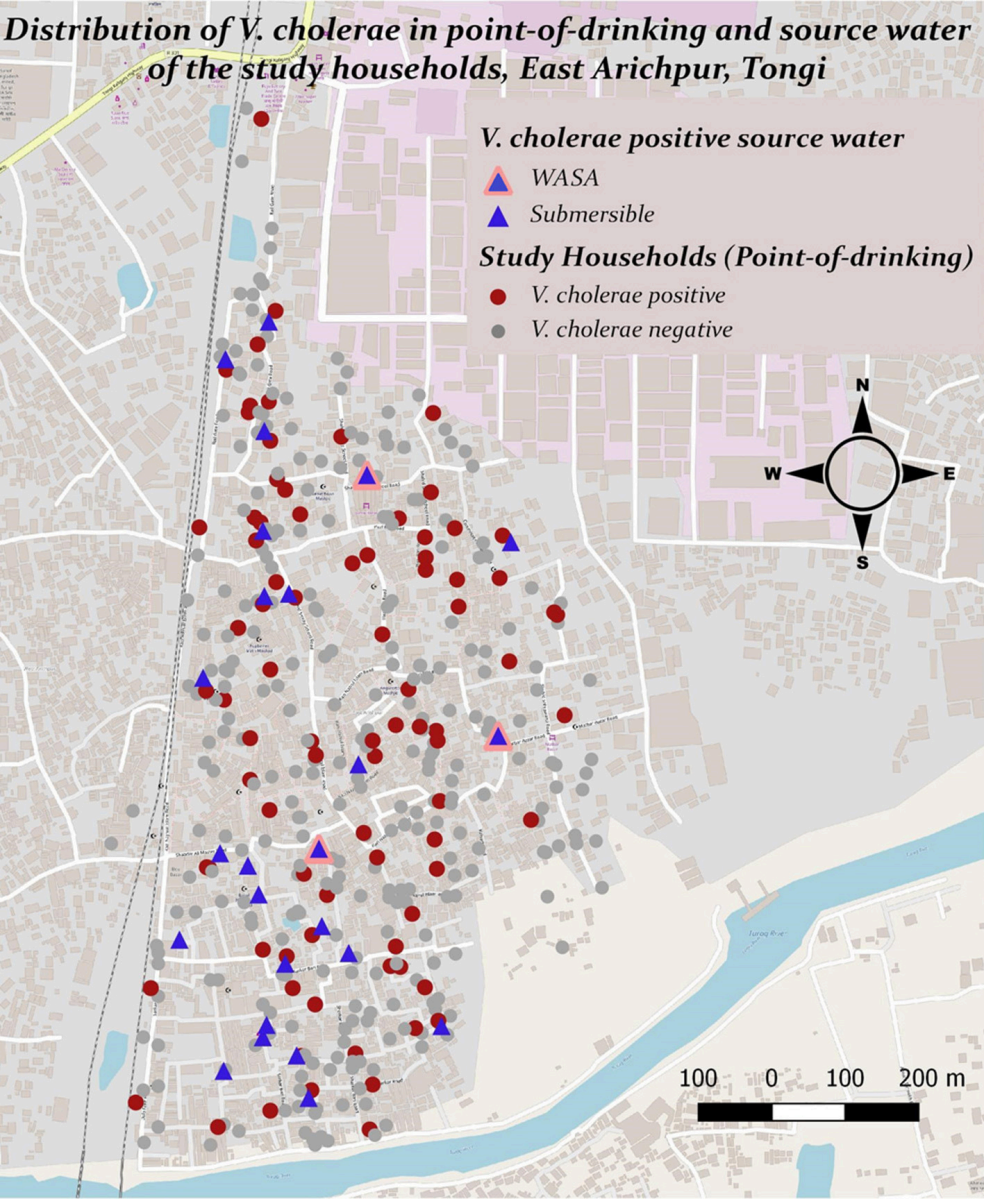
Presence and distribution of *V. cholerae* in point-of-drinking and source water samples from the study households in Arichpur, Bangladesh, September 2014–October 2015.

*V. cholerae* was detected in 10% (110/1082) of point-of-drinking water samples and in 9% (33/381) of source water samples (Table 1). Point-of-drinking water from 23% of households (88/388) and source water for 38% (25/66) of households were positive for *V. cholerae* at least once in the visits conducted at 6 week intervals. Most (76%, 67/88) households with point-of-drinking water samples positive for *V. cholerae* were also connected to 19 of 25 *V. cholerae* positive sources, irrespective of timing of collection. However, from the stratified data in 7 day intervals, 53% (17/32, [(95% CI = 0.360–0.70)]) of the sources were negative for *V. cholerae*, while point-of-drinking water samples linked to these sources were positive. The percentage of samples positive for *V. cholerae* was higher in the point-of-drinking water (11% [32/299], *P* = 0.000) compared to the sources water (9%, 28/299) in the 7 day-interval stratified data. The *V. cholerae* positive households were distributed throughout the study area, whereas the *V. cholerae* positive sources were mainly clustered in the southern part of the study area, which is adjacent to a water body (Figure 1).

**Table 1:** Presence of *V. cholerae* in point-of-drinking and source water samples from the study households in Arichpur, Bangladesh, September 2014–October 2015.

**Characteristics of point-of-drinking water**	**Sample**	
	**N (%)**	***V. cholerae* positive n (%)**
	**[*N* = 1082]**	**[*n* = 110]**
Treated water	165 (15)	20 (12)
Non-treated water	917 (85)	90(10)
**Types of treatment carried out**	**[*****n*** = **165]**	
Boiling	125 (76)	14 (13)
Filtration	31 (19)	4 (4)
Boiling and filtration	4 (2)	2 (2)
**Types of drinking vessels used at the point-of-drinking**	**[*****n*** = 1069]^*^	
Mug	575 (54)	52 (47)
Glass	334 (31)	41 (37)
Bottle	125 (12)	8 (7)
Jug	30 (3)	6 (6)
Pitcher	5 (1)	1 (1)
**Characteristics of source water**	**N (%) [*****N*** = **381]**	**n (%) [*****n*** = **33]**
**By types of collection points**		
Taps attached to the communal pumps	146 (38)	6 (15)
Taps attached to the reservoir connected to the pumps	235 (62)	27 (82)
**By types of pumps**		
WASA pump	36 (9)	4 (12)
Submersible pump	345 (91)	29 (88)

* *For some samples, the types of vessels used were not known*.

In point-of-drinking water, *V. cholerae* was detected twice in 15% (13/88) of households, three times in 2% (2/88) of households, and six times in 1% (1/88) of households. The probability of the presence of *V. cholerae* was higher in glasses than mugs and bottles (Table 2). There was a higher probability of the presence of *V. cholerae* in non-treated water compared to treated water (*P* = 0.22; Table 2). *V. cholerae* was detected twice in 8% (2/25) of sources, three times in 4% (1/25) of sources, and five times in 4% (1/25) of sources. Samples from all three WASA pumps' were positive for *V. cholerae* at least once (Figure 1). Most (82%, 29/33) of the *V. cholerae* detected in source water came from taps attached to the reservoir connected to the pumps (*P* = 0.008), rather the taps directly attached to the communal pumps (Table 2).

**Table 2:** Logistic regression of factors associated with the presence of *V. cholerae* in water samples from Arichpur, Bangladesh, September 2014–October 2015.

**Factors**	**OR (95% CI)**	**P**
**PRESENCE OF** ***V. cholerae***		
Point-of-drinking vs. source (irrespective of the timing of sample collection)	1.19 (0.79–1.79)	0.230
Point-of-drinking vs. source (samples collected within 7 day interval)	17.24 (7.14–42.89)	0.000^*^
Taps attached to the reservoir connected to the pumps vs. taps attached to the communal pumps	3.03 (1.22–7.53)	0.008^*^
Non-treated vs. treated point-of-drinking water	1.27 (0.76–2.12)	0.220
Point-of-drinking water in glass vs. mug	1.41 (0.91–2.17)	0.076
Glass vs. bottle	2.05 (0.93–4.50)	0.046
Mug vs. bottle	1.45 (0.67–3.14)	0.221
**PRESENCE OF TOXIGENIC** ***V. cholerae*** **O1/O139**		
Source vs. point-of-drinking	6.22 (2.54–15.25)	0.000^*^
Taps attached to the reservoir connected to the pumps vs. taps attached to the communal pumps	1.74 (0.55–5.58)	0.254

OR, odds ratio; CI, confidence interval;

* *significance at a level of P ≤ 0.05*.

### Distribution of virulence genes

A total of 143 *V. cholerae* positive samples were identified, and virulence genes other than *ompW* were detected in these positive samples. In total, 11% (15/143) of *V. cholerae* samples were positive for the *rfb* O1 gene and 6% (9/143) of *V. cholerae* samples were positive for the *rfb* O139 gene (Table 3). The percentages of serogroups O1 and O139 were higher in source water compared to point-of-drinking water. There was a higher probability of having *V. cholerae* O1 [OR = 9.13 (95% CI = 2.85–29.26)] and *V. cholerae* O139 [OR = 4.73 (95% CI = 1.19–18.79)] in source water compared to point-of-drinking water (Table 3). Of the samples with non-O1/non-O139 serogroups, the *ctxA* gene was found in three of the point-of-drinking water samples and two of the source samples. The percentage of samples in which the *hlyA* gene was detected was higher in point-of-drinking water compared to source water, and this difference was statistically significant (Table 3).

**Table 3:** Presence and logistic regression of *V. cholerae* virulence genes in source and point-of-drinking water samples from Arichpur, September 2014–October 2015.

**Genes**	**Total samples positive for *V. cholerae* n = 143, n (%)**	**Point-of-drinking water samples positive for *V. cholerae* n = 110, n (%)**	**Source water samples positive for *V. cholerae* n = 33, n (%)**	**Odds ratio of source vs. point-of-drinking water samples (95% CI)**	***P*-value**
*ctxA*	8 (6)	7 (5)	4 (9)	2.10 (0.47–9.30)	0.271
*rfbO1*	15 (11)	5 (5)	10 (31)	9.13 (2.85–29.26)	0.000^*^
*rfbO139*	9 (6)	4 (4)	5 (16)	4.73 (1.19–18.79)	0.031^*^
*cep*	62 (44)	50 (46)	12 (38)	0.67 (0.31–1.53)	0.235
*ace*	7 (5)	5 (5)	2 (6)	1.36 (0.25–7.32)	0.510
*msh1*	45 (32)	34 (31)	11 (34)	1.12 (0.49–2.56)	0.475
*stn/sto*	9 (6)	3 (3)	6 (19)	7.93 (1.86–33.75)	0.005^*^
*rtxA*	36 (25)	24 (22)	12 (38)	2.05 (0.88–4.75)	0.075
*toxR*	97 (68)	77 (70)	20 (63)	0.66 (0.29–1.48)	0.210
*tcpI*	3 (2)	2 (2)	1 (3)	1.69 (0.15–19.22)	0.548
*hlyA*	121 (85)	99 (90)	22 (69)	0.22 (0.09–0.58)	0.002^*^
*ompU*	10 (7)	7 (6)	3 (9)	1.47 (0.36–6.04)	0.417
*nag-st*	3 (2)	3 (3)	0 (0)	–	–
*rtxC*	61 (43)	47 (43)	14 (44)	0.98 (0.45–2.17)	0.569
*hap*	88 (62)	70 (64)	18 (56)	0.69 (0.31–1.51)	0.229
*chxA*	56 (39)	39 (36)	17 (53)	1.93 (0.88–4.24)	0.074
*vcsC2*	8 (6)	6 (5)	2 (6)	1.11 (0.22–5.82)	0.589
*vcsN2*	8 (6)	6 (5)	2 (6)	1.11 (0.22–5.82)	0.589
*vopF*	12 (8)	9 (8)	3 (9)	1.12 (0.29–4.41)	0.554
*vasK*	141 (99)	109 (99)	32 (100)	–	–
*vasA*	140 (99)	109 (99)	31 (97)	0.14 (0.01–1.62)	0.133
*vasH*	140 (99)	109 (99)	31 (97)	0.14 (0.01–1.62)	0.133

* *P ≤ 0.05*.

Two of the *V. cholerae* positive point-of-drinking water samples carried virulence genes- *ctxA*, as well as, *rtxA, rtxC, toxR, hlyA, hap, msh1, chxA*, T6SS but lacked *tcpI, ompU, ace, nag-st. One* point-of-drinking water sample was found positive for *hlyA, rtxA, toxR, hap, ompU, cep, chxA*, and T6SS, but negative for *ctxA, rtxC, tcp*, and *ace*. One of the source water samples exhibited *hlyA, tcp, hap, cep, mshA, chxA*, T3SS, and T6SS, but not *ctxA, rtxA*, or *ompU*. However, most of the 121 non-O1/O139 *V. cholerae* positive samples carried *hlyA, rtxA, hap*, and *toxR*, as well as genes encoding T6SS.

## Discussion

Toxigenic and non-toxigenic *V. cholerae* were widely distributed in point-of-drinking and source waters throughout the low-income urban community of Arichpur. The estimated probability of the presence of *V. cholerae* in point-of-drinking water when absent in linked sources was 0.53 (95% CI = 0.36–0.70) within 7 day intervals, which suggests that post-contamination of point-of-drinking water might have occurred. The probability of the presence of *V. cholerae* O1 [OR = 9.13 (95% CI = 2.85–29.26)] and O139 [OR = 4.73 (95% CI = 1.19–18.79)] in source water was significantly higher than that in the point-of-drinking water, suggesting that the quality of point-of-drinking water might not be affected by the quality of sources.

Similar to other studies (Wright et al., 2004; Rufener et al., 2010), our study showed that the contamination of water was higher at the point-of-drinking compared to the source. In an observational study in Pakistan, Jensen et al. (2002) showed that water stored inside the household was more often contaminated than the source water when the source water contained < 100 *E. coli* per 100 mL (Jensen et al., 2002). In this same study, the researchers performed a 5 week intervention using narrow-necked water pitcher (that prevent utensils or hands from retrieving water) to prevent water contamination and found a significant improvement in in-house water quality (Jensen et al., 2002). A systematic review indicated that water quality improvement at sources were ineffective, because water from a good quality source was often contaminated at the point of use through poor hygiene practices in households (Taylor et al., 2015). Another study conducted in Bolivia showed that pathogen-free water at the source is not a guarantee for safe and pathogen-free drinking water at the point-of-consumption (Rufener et al., 2010), supporting our findings that the quality of water at the point-of-drinking did not depend on the presence or absence of *V. cholerae* in the source water.

Although there are reports that treatment type (boiling, chlorination) (Momba and Notshe, 2003; Levy et al., 2008), have significant impact on drinking water quality, our study did not evidence any significant association of *V. cholerae* with specific treatment type (boiling or filtration). A study conducted at the household level in rural areas of Peru reported that 69% of jars in which drinking water was stored had fecal coliforms though the water was treated by boiling (Gil et al., 2014). The absence of a holding shaft on a glass might play role in reducing direct hand contamination of drinking water to some extent and might explain the higher probabilities of *V. cholerae* contamination of water in drinking glasses compared to mugs. Compared to glasses and mugs, bottles were less frequently contaminated with *V. cholerae*, suggesting that narrow-necked vessels can prevent contamination, as shown by Jensen et al. (2002).

The higher prevalence of toxigenic *V. cholerae* O1/O139 [OR = 6.22 (2.54–15.25)] in sources compared to point-of-drinking water in this study matched findings of other studies conducted in Dhaka (Rafique et al., 2016) and in northern coastal Ecuador (Levy et al., 2008). In the source water, the number of *V. cholerae* was significantly higher in the water samples collected from the taps attached to the reservoir connected to the pumps compared to taps attached to the communal pumps which was also in agreement with a study conducted in Ecuador (Chalchisa et al., 2017). Larger storage tanks allowing longer storage times without regular cleaning (Schafer and Mihelcic, 2012) may potentially increase the risk of contamination and allow the persistence of bacteria by inducing the VBNC state (Colwell et al., 1996; Thomas et al., 2006).

*V. cholerae* lacking the *tcpI* gene was found in 5% *ctxA* positive samples. This is consistent with results of a study in Bangladesh (Hasan et al., 2013), where environmental O1 toxigenic strains were found to lack the *tcpA* and *tcpI* genes. Furthermore, we obtained O1 positive samples that did not carry *ctxA, tcpI* but carried *hlyA, hap, rtxA* genes. A research showed that variant virulence profile can be observed, since environmental strains are more heterogeneous than clinical strains (Hasan et al., 2013).

We found that non-O1/non-O139 *V. cholerae* was widely distributed throughout both source and point-of-drinking water samples. These strains are recognized to be of public health relevance, because they have been associated with sporadic cases or outbreaks of cholera-like disease (Crump et al., 2003; Dutta, 2013) and many extra-intestinal infections (Akoachere and Mbuntcha, 2014). While it is true that most epidemic cholera cases are caused by toxigenic *V. cholerae* O1/O139, a large proportion of diarrheal cases do not have a defined etiology where surveys take place (Islam et al., 2013).

After analyzing the genetic profiles of *V. cholerae* in samples, 85% of the *V. cholerae* in positive samples possessed *hlyA*, a gene whose product is an exotoxin related to CT, and *rtxA*, a heat-stable enterotoxin, both of which can be found in non-O1 strains isolated from patients with cholera (Saka et al., 2008) and from environmental strains from endemic areas (Faruque et al., 2004; Kumar et al., 2008; Mohapatra et al., 2009). These samples also possessed toxR, a 32-kDa transmembrane protein that acts as a master regulator of the *ctxAB* gene (DiRita et al., 1991). Finally, 10% of *V. cholerae* positive samples possessed *ompU*, whose product has been implicated in colonization and can also be found in some environmental isolates from endemic regions (Karunasagar et al., 2003). A gene *mshA*, also implicated in colonization, encoding a type IV pilus and biofilm formation on abiotic (borosilicate glass) and biotic surfaces (cellulose) (Watnick et al., 1999), was present in approximately half of the *V. cholerae* positive samples. This might explain the higher frequency of *V. cholerae* detection in the reservoir tanks.

Recently, fatal diarrheal disease caused by non-O1/O139 strains of *V. cholerae* has been shown to be associated with T3SS (Tam et al., 2010; Shin et al., 2011), a system absent in common pandemic O1 strains. Six percent of *V. cholerae* drinking water samples were positive for the presence of T3SS, implying the potential to cause fatal diarrhea via drinking water. Two other genes, *chxA* (encoding cholix toxin) was present in 39% and hap was present in 62% of the samples, which are also known to be associated with virulence in non-pandemic strains (Islam et al., 2013).

Our study had some limitations. Data presented here did not consider the inclusion of isolates. However, PCR performed directly on DNA samples allowed us to detect toxigenic genes of *V. cholerae* in both the culturable and non-culturable state, the latter of which can explain cholera or cholera-like diarrheal illness resulting from drinking water. In addition, PCR provides rapid detection with reduced cost compared to the culture method, and so might be useful for identifying the pathogen in outbreak settings. Although we found significantly higher presence of toxigenic *V. cholerae* O1/O139 in source water compared to point-of-drinking water, this assumption should be interpreted carefully, since the number of samples is low in this study.

## Conclusion

Our study findings showed that contamination of water at point-of-drinking was less likely to depend on the contamination at sources and presence of *V. cholerae* in point-of-drinking water possibly did not depend on home-based water treatment suggesting that different routes (by hand, drinking vessel, flies) might have facilitated the contamination of drinking water at point-of-drinking. Hygiene education intervention and program should focus and emphasize on point-of-drinking including repeated cleaning of drinking vessels (such as mug, glass, bottle), which is of paramount importance in the prevention of cholera and cholera-like diarrheal illness. Data obtained in our study will serve as the baseline for the future investigations of *V. cholerae* in the environment, particularly in water.

## Author contributions

JF designed the study concept, conducted the study in the laboratory, performed statistical analysis, and wrote the manuscript. RS contributed to framing the manuscript, data analysis, and writing and critical revision of the manuscript. RR contributed to the study concept, laboratory work, and data acquisition. MT contributed to the laboratory work and data acquisition. AN performed part of the statistical analysis. AB was the principal investigator of the project, contributed reagents, and approved the final version of the manuscript to be submitted. PJ was the functional principal investigator of the project and contributed to manuscript development, critical revision, and approval of the final version to be published.

### Conflict of interest statement

The authors declare that the research was conducted in the absence of any commercial or financial relationships that could be construed as a potential conflict of interest.
