# Supplementary material for: A Comparative Analysis of Vibrio cholerae Contamination in Point-of-Drinking and Source Water in a Low-Income Urban Community, Bangladesh
Source: Front Microbiol. 2018 Mar 19;9:489. doi: 10.3389/fmicb.2018.00489 (PMC5867346; doi:10.3389/fmicb.2018.00489)
Supplement: Supplementary file 1 [file Table1.docx]

Supplementary Material

**A comparative analysis of *Vibrio cholerae* contamination in point-of-drinking and source water in a low-income urban community, Bangladesh**

Jannatul Ferdous^1, 2^, Rebeca Sultana^2, 3, 4^, Ridwan Bin Rashid^1^, Md. Tasnimuzzaman^1^, Andreas Nordland^2^, Anowara Begum^1^, Peter Kjaer Mackie Jensen^2^

^1^Department of Microbiology, University of Dhaka, Dhaka, Bangladesh

^2^Copenhagen Centre for Disaster Research, Section for Global Health, Department of Public Health, University of Copenhagen, Copenhagen, Denmark

^3^ icddr,b, Dhaka, Bangladesh

^4^ Institute of Health Economics, University of Dhaka, Dhaka, Bangladesh

* **Correspondence**:

Jannatul Ferdous

jannat.du2010@gmail.com

jannatul@sund.ku.dk

**S1 Table: Target genes for amplicon sizes and references for virulence gene detection**

| **Target genes** | | **Primer** | **Sequence (5′-3′)** | **Annealing (^°^C)** | | **Amplicon sizes (bp)** | **References** |
| --- | --- | --- | --- | --- | --- | --- | --- |
| ***ompW*** | ompW-F | | CAC CAA GAA GGT GAC TTT ATT GTG | | 57 | 304 | (Nandi et al., 2000) |
|  | ompW-R | | GGT TTG TCG AAT TAG CTT CAC C | |  |  |  |
| ***toxR*** | tox-F | | CCT TCG ATC CCC TAA GCA ATAC | | 53 | 779 | (Rivera et al., 2001) |
|  | tox-R | | AGG GTT AGC AAC GAT GCG TAA G | |  |  |  |
| ***tcpI*** | tcpI-F | | TAG CCT TAG TTC TCA GCA GGC A | | 52 | 862 | (Rivera et al., 2001) |
|  | tcpI-R | | GGC AAT AGT GTC GAG CTC GTT A | |  |  |  |
| ***rfb* O1** | rfb O1-F | | TCT ATG TGC TGC GAT TGG TG | | 57 | 638 | (Goel et al., 2007) |
|  | rfb O1-R | | CCC CGA AAA CCT AAT GTG AG | |  |  |  |
| ***rfb*-O139** | rfb-O139-f | | AGC CTC TTT ATT ACG GGT GG | | 57 | 449 | (Hoshino et al., 1998) |
|  | rfb-O139-r | | GTC AAA CCC GAT CGT AAA GG | |  |  |  |
| ***ace*** | ace-F | | TAA GGA TGT GCT TAT GAT GGA CAC CC | | 52 | 309 | (Hoshino et al., 1998) |
|  | ace-R | | CGT GAT GAA TAA AGA TAC TCA TAG G | |  |  |  |
| ***cep*** | cep-F | | GCT ACA TGT TTA GCT CAC TG | | 48 | 251 | (Bhattacharya et al., 2006) |
|  | cep-R | | TTT AGC CTT ACG AAT TAA GCC | |  |  |  |
| ***hlyA*** | hlyA-F | | GAG CCG GCA TTC ATC TGA AT | | 51 | 480 | (Kumar et al., 2009) |
|  | hlyA-F | | CTC AGC GGG CTA ATA CGG TTT A | |  |  |  |
| ***ompU*** | ompU-F | | CCA AAG CGG TGA CAA AGC | | 48 | 655 | (Kumar et al., 2009) |
|  | ompU-R | | TTC CAT GCG GTA AGA AGC | |  |  |  |
| **NAG-ST** | NAG-ST-F | | CCT ATT CAT TAG CAT AAT G | | 42 | 215 | (Chatterjee et al., 2009) |
|  | NAG-ST-R | | CCA AAG CAA GCT GGA TTG C | |  |  |  |
| ***stn/sto*** | stn/sto-F | | TCG CAT TTA GCC AAA CAG TAG AAA | | 51 | 172 | (Rivera et al., 2001) |
|  | stn/sto-R | | GCT GGA TTG CAA CAT ATT TCG C | |  |  |  |
| ***rtxC*** | rtxC-F | | CGA CGA AGA TCA TTG ACG AC | | 51 | 265 | (Chow et al., 2001) |
|  | rtxC-R | | CAT CGT CGT TAT GTG GTT GC | |  |  |  |
| ***msh1*** | msh1-F | | AAA AGT CGA CAG CGA AAG CGA ATA GTG G | | 60 | 380 | (Thelin and Taylor, 1996) |
|  | msh2-R | | AAA AGG ATC CAT TGC ACC AGC AAC TGC ACC | |  |  |  |
| ***hap*** | HA/P-F | | ACG TTA GTG CCC ATG AGG TC | | 49 | 350 | (Haley et al., 2012) |
|  | HA/P-R | | ACG GCA AAC ACT TCA AAA CC | |  |  |  |
| ***chxA*** | chxA- F | | TGG TGA AGA TTC TCC TGC AA | | 49 | 421 | (Jørgensen et al., 2008) |
|  | chxA-R | | CTT GGA GAA ATG GAT GCG CTG | |  |  |  |
| ***vcsC2 (TTSS)*** | vcsC2-F | | GGT CTC ATA GAC ACT ACG | | 48 | 589 | (Awasthi et al., 2013) |
|  | vcsC2-R | | ACG ATG CTA TGG GGT ATG | |  |  |  |
| ***vcsN2(TTSS)*** | vcsN2-F | | CAA CAC CTT CAA AGC CTT G | | 48 | 848 | (Awasthi et al., 2013) |
|  | vcsN2-R | | GCG AGC TCC AAT TGA AAC | |  |  |  |
| ***vopF(TTSS)*** | vopF-U | | GGA AAT TCG CCA AGG TGT A | | 48 | 839 | (Awasthi et al., 2013) |
|  | vopF-R | | CAA AAC CGT CCAT ACA AGG | |  |  |  |
| ***vasH (T6SS)*** | vasH-857F | | GTG GCA CGC TAT TTC TGG AT | | 49 | 385 | (Hasan et al., 2013) |
|  | vasH-1242R | | TTTCAGCTCACGCACATTTC | |  |  |  |
| ***vasA(T6SS)*** | vasA-104F | | GTA CGA CCG ATC CTG ACG TT | | 51 | 342 | (Hasan et al., 2013) |
|  | vasA-446R | | ATC TGA ATG GTC GTG GCT TC | |  |  |  |
| ***vasK(T6SS)*** | vasK-1851F | | GCG TCA AAT TCA GGA AGA GC | | 51 | 399 | (Hasan et al., 2013) |
|  | vasK-2250R | | CTG TCC CAG AAC CCA ACT GT | |  |  |  |

**References**

Awasthi, S.P., Asakura, M., Chowdhury, N., Neogi, S.B., Hinenoya, A., Golbar, H.M., et al. (2013). Novel cholix toxin variants, ADP-ribosylating toxins in Vibrio cholerae non-O1/non-O139 strains, and their pathogenicity. *Infection and immunity* 81(2)**,** 531-541. doi: 10.1128/IAI.00982-12.

Bhattacharya, T., Chatterjee, S., Maiti, D., Bhadra, R.K., Takeda, Y., Nair, G.B., et al. (2006). Molecular analysis of the rstR and orfU genes of the CTX prophages integrated in the small chromosomes of environmental Vibrio cholerae non‐O1, non‐O139 strains. *Environmental microbiology* 8(3)**,** 526-634. doi: 10.1111/j.1462-2920.2005.00932.x.

Chatterjee, S., Ghosh, K., Raychoudhuri, A., Chowdhury, G., Bhattacharya, M., Mukhopadhyay, A., et al. (2009). Incidence, virulence factors, and clonality among clinical strains of non-O1, non-O139 Vibrio cholerae isolates from hospitalized diarrheal patients in Kolkata, India. *Journal of clinical microbiology* 47(4)**,** 1087-1095. doi: 10.1128/JCM.02026-08.

Chow, K., Ng, T., Yuen, K., and Yam, W. (2001). Detection of RTX toxin gene in Vibrio cholerae by PCR. *Journal of clinical microbiology* 39(7)**,** 2594-2597. doi: 10.1128/JCM.39.7.2594-2597.2001.

Goel, A., Ponmariappan, S., Kamboj, D., and Singh, L. (2007). Single multiplex polymerase chain reaction for environmental surveillance of toxigenic—Pathogenic O1 and Non-O1 vibrio cholerae. *Folia microbiologica* 52(1)**,** 81-85. doi: org/10.1007/BF02932143.

Haley, B.J., Chen, A., Grim, C.J., Clark, P., Diaz, C.M., Taviani, E., et al. (2012). Vibrio cholerae in a historically cholera‐free country. *Environmental microbiology reports* 4(4)**,** 381-389. doi: 10.1111/j.1758-2229.2012.00332.x.

Hasan, N.A., Ceccarelli, D., Grim, C.J., Taviani, E., Choi, J., Sadique, A., et al. (2013). Distribution of virulence genes in clinical and environmental Vibrio cholerae strains in Bangladesh. *Applied and environmental microbiology* 79(18)**,** 5782-5785. doi: 10.1128/AEM.01113-13.

Hoshino, K., Yamasaki, S., Mukhopadhyay, A.K., Chakraborty, S., Basu, A., Bhattacharya, S.K., et al. (1998). Development and evaluation of a multiplex PCR assay for rapid detection of toxigenic Vibrio cholerae O1 and O139. *FEMS Immunology & Medical Microbiology* 20(3)**,** 201-207. doi: 10.1111/j.1574-695X.1998.tb01128.x.

Jørgensen, R., Purdy, A.E., Fieldhouse, R.J., Kimber, M.S., Bartlett, D.H., and Merrill, A.R. (2008). Cholix toxin, a novel ADP-ribosylating factor from Vibrio cholerae. *Journal of Biological Chemistry* 283(16)**,** 10671-10678. doi: 10.1074/jbc.M710008200.

Kumar, P., Jain, M., Goel, A., Bhadauria, S., Sharma, S., Kamboj, D., et al. (2009). A large cholera outbreak due to a new cholera toxin variant of the Vibrio cholerae O1 El Tor biotype in Orissa, Eastern India. *Journal of medical microbiology* 58(2)**,** 234-238. doi: 10.1099/jmm.0.002089-0.

Nandi, B., Nandy, R.K., Mukhopadhyay, S., Nair, G.B., Shimada, T., and Ghose, A.C. (2000). Rapid method for species-specific identification ofVibrio cholerae using primers targeted to the gene of outer membrane protein OmpW. *Journal of clinical microbiology* 38(11)**,** 4145-4151. doi: <https://www.ncbi.nlm.nih.gov/pmc/articles/PMC87555/>.

Rivera, I.N., Chun, J., Huq, A., Sack, R.B., and Colwell, R.R. (2001). Genotypes associated with virulence in environmental isolates of Vibrio cholerae. *Applied and Environmental Microbiology* 67(6)**,** 2421-2429. doi: 10.1128/AEM.67.6.2421-2429.2001.

Thelin, K.H., and Taylor, R.K. (1996). Toxin-coregulated pilus, but not mannose-sensitive hemagglutinin, is required for colonization by Vibrio cholerae O1 El Tor biotype and O139 strains. *Infection and immunity* 64(7)**,** 2853-2856. doi: <https://www.ncbi.nlm.nih.gov/pmc/articles/PMC174155/pdf/642853.pdf>

.
