# Supplementary material for: A Comparative Analysis of Vibrio cholerae Contamination in Point-of-Drinking and Source Water in a Low-Income Urban Community, Bangladesh
Source: Front Microbiol. 2018 Mar 19;9:489. doi: 10.3389/fmicb.2018.00489 (PMC5867346; doi:10.3389/fmicb.2018.00489)
Supplement: Supplementary file 2 [file Table2.docx]

Supplementary Material

**A comparative analysis of *Vibrio cholerae* contamination in point-of-drinking and source water in a low-income urban community, Bangladesh**

Jannatul Ferdous^1, 2^, Rebeca Sultana^2, 3, 4^, Ridwan Bin Rashid^1^, Md. Tasnimuzzaman^1^, Andreas Nordland^2^, Anowara Begum^1^, Peter Kjaer Mackie Jensen^2^

^1^Department of Microbiology, University of Dhaka, Dhaka, Bangladesh

^2^Copenhagen Centre for Disaster Research, Section for Global Health, Department of Public Health, University of Copenhagen, Copenhagen, Denmark

^3^ icddr,b, Dhaka, Bangladesh

^4^ Institute of Health Economics, University of Dhaka, Dhaka, Bangladesh

* **Correspondence**:

Jannatul Ferdous

jannat.du2010@gmail.com

jannatul@sund.ku.dk

**S2 Table: Target genes for qPCR, qPCR primers and probe, amplicon sizes and references**

| **Target genes** | **Primer (5′-3′)** | | **Amplicon sizes (bp)** | **Assays** | **References** |
| --- | --- | --- | --- | --- | --- |
| ***ctxA*** | Sense (ctxA-F) | TTT GTT AGG CAC GAT GAT GGA | 84 | TaqMan MasterMix | (Blackstone et al., 2007) |
|  | Antisense (ctxA-R) | ACC AGA CAA TAT AGT TTG ACC CAC TAA |  |  |  |
|  | Probe (ctxA-P) | Fam-TGT TTC CAC CTC AAT TAG TTT GAG AAG TGC CC-BHQ-1 |  |  |  |
| ***rtxA*** | Sense (rtxA-v1F) | AGC AAG AGC ATT GTT GTT CCT ACC | 120 | SYBR Green MasterMix | (Gubala, 2006) |
|  | Antisense (rtxA-v1R) | ACT TCC CTG TAC CGC ACT TAG AC |  |  |  |

**Reference**

Blackstone, G.M., Nordstrom, J.L., Bowen, M.D., Meyer, R.F., Imbro, P., and DePaola, A. (2007). Use of a real time PCR assay for detection of the ctxA gene of Vibrio cholerae in an environmental survey of Mobile Bay. *Journal of Microbiological Methods* 68(2)**,** 254-259. doi: 10.1016/j.mimet.2006.08.006.

Gubala, A.J. (2006). Multiplex real-time PCR detection of Vibrio cholerae. *Journal of microbiological methods* 65(2)**,** 278-293. doi: 10.1016/j.mimet.2005.07.017.
